# Combining Support and Assessment in Health Professions Education: Mentors’ and Mentees’ Experiences in a Programmatic Assessment Context

**DOI:** 10.5334/pme.1004

**Published:** 2023-07-07

**Authors:** Lianne M. Loosveld, Erik W. Driessen, Mattias Theys, Pascal W. M. Van Gerven, Eline Vanassche

**Affiliations:** 1 Department of Educational Development & Research, School of Health Professions Education, Faculty of Health, Medicine and Life Sciences, Maastricht University, Universiteitssingel 60, 6229 ER Maastricht, the Netherlands; 2 Faculty of Psychology and Educational Sciences, KU Leuven Kulak, Etienne Sabbelaan 51, P.O. Box 7654, 8500 Kortrijk, Belgium

## Abstract

**Introduction:**

Mentors in programmatic assessment support mentees with low-stakes feedback, which often also serves as input for high-stakes decision making. That process potentially causes tensions in the mentor-mentee relationship. This study explored how undergraduate mentors and mentees in health professions education experience combining developmental support and assessment, and what this means for their relationship.

**Methods:**

The authors chose a pragmatic qualitative research approach and conducted semi-structured vignette-based interviews with 24 mentors and 11 mentees that included learners from medicine and the biomedical sciences. Data were analyzed thematically.

**Results:**

How participants combined developmental support and assessment varied. In some mentor-mentee relationships it worked well, in others it caused tensions. Tensions were also created by unintended consequences of design decisions at the program level. Dimensions impacted by experienced tensions were: relationship quality, dependence, trust, and nature and focus of mentoring conversations. Mentors and mentees mentioned applying various strategies to alleviate tensions: transparency and expectation management, distinguishing between developmental support and assessment, and justifying assessment responsibility.

**Discussion:**

Combining the responsibility for developmental support and assessment within an individual worked well in some mentor-mentee relationships, but caused tensions in others. On the program level, clear decisions should be made regarding the design of programmatic assessment: what is the program of assessment and how are responsibilities divided between all involved? If tensions arise, mentors and mentees can try to alleviate these, but continuous mutual calibration of expectations between mentors and mentees remains of key importance.

## Introduction

Undergraduate mentors in health professions education (HPE) are increasingly involved in the programmatic assessment of their mentees, wherein multiple low-stakes assessments are aggregated to serve as robust input for high-stakes decision making (e.g., receiving course credits or not, passing an entire year, go/no-go decisions on progression to clinical rotations). Mentors can take different approaches to support the personal and professional development of mentees in health professions education (HPE). They can, for example, act as a role model to foster professional behavior, ask questions to stimulate reflection on past performance, support mentees in building a portfolio, help with interpreting feedback from others, and provide feedback themselves [1,2,3,4,5,6,7,8,9]. In doing so, mentors may unintentionally merge supportive feedback with evidence for performance-based decision making [10,11,12,13,14,15,16].

Several authors [9,16,17,18,19] conclude that tensions arise when feedback intended to support the growth of the learner is also used as input for high-stakes decision making such as pass/fail assessments. In some residency programs, where the mutual relationship between the supervisor and the learner could be considered similar to that between an undergraduate mentor and mentee, using feedback for this dual purpose led to changing dynamics between the learner and the supervisor, lower quality of feedback, and increased difficulty for learners to discriminate between low- and high-stakes assessments [17]. Moreover, it made learners change their behavior to please their supervisor, hide vulnerabilities, or avoid seeking feedback on certain aspects of their functioning altogether [9,12,20]. Thus, no matter what the assessment intentions were, learners tend to perceive low-stakes feedback as a high-stakes assessment [9,11,17,21,22].

Furthermore, tensions are not only experienced by learners, but by their teachers and supervisors as well [11,12,20,23]. Especially for mentors, being perceived as both a provider of developmental support and an assessor could have detrimental effects on the mentor-mentee relationship [24,25,26]. In a study by Schut et al. [11], for example, teachers indicated that they refrained from building close relationships with their students in order to minimize potential personal bias during the assessment process. Purposefully creating distance might hinder a trusting mentor-mentee relationship.

Earlier work on the personal interpretative framework [27] of mentors [24,25] has demonstrated that mentors actively shape their mentoring practice based on the interaction between their knowledge and beliefs about mentoring, and the context within which they operate. This in turn determines what, to them, are valuable goals and purposes of mentoring. However, conflicting narratives may arise when mentors’ task perception and their definition of what it means to be a mentor are misaligned with program requirements such as having to assess mentees. This potentially inhibits mentors from putting their personal knowledge and beliefs about mentoring into practice, with adverse effects [28,29,30], such as impacting their self-esteem and future motivation for mentoring [29].

Based on these observations, we argue that entrusting mentors with the support of mentees while also being involved in their programmatic assessment potentially causes tensions for both. Therefore, we investigated what combining these responsibilities means for the mentoring relationship, which is often characterized by open, honest, and, at times, sensitive conversations between mentors and mentees. For this purpose, we interviewed both mentors and mentees in HPE with the following research question in mind: How do undergraduate mentors and mentees experience combining developmental support and assessment in a programmatic assessment context?

## Methods

### Design

Because we aimed to describe and understand how undergraduate mentors and mentees experience combining developmental support and assessment, we used a pragmatic qualitative research approach and thematic analysis [31]. We worked from a constructivist philosophical perspective, acknowledging and aiming to understand mentors’ and mentees’ experiences in and of mentoring, and how they actively make sense of these experiences in interaction with a particular program context [32,33].

### Setting

We purposefully selected undergraduate (pre-clinical) programs from the Faculty of Health, Medicine and Life Sciences at Maastricht University in the Netherlands. We selected those programs in which mentors support mentees’ personal and professional development, and were involved in their portfolio-based programmatic assessment. Two programs met these criteria: Medicine and Biomedical Sciences at the Faculty of Health Medicine and Life Sciences.

In both programs, mentors support mentees for the entire three-year duration of their undergraduate program. They support groups of five (Medicine) or nine to 16 mentees (Biomedical Sciences), with whom they meet three to five times a year, both individually and in groups. Mentor-mentee dyad allocations were assigned randomly within the respective programs. Individual meetings focus on development goals formulated by the mentees, and are often based on evidence mentees gather on so-called “reflection cards” in their e-portfolio [34,35]. Mentors do not observe their mentees in educational or clinical settings. In both programs, mentors are involved in the programmatic assessment process: at the end of each academic year, they are requested to give a pass/fail advice [22]. Mentors base their advice on the meetings they had with mentees throughout the year and the information mentees gathered in their portfolios (e.g., reflections on experiences, action plans, self-directed learning diaries, progress test results).

Mentors collaborate in that decision making process with a second mentor from the same program with whom they can discuss mentees’ progress. In both programs, this ‘second pair of eyes’ [35] also checks mentees’ portfolios and endorses or challenges mentors’ end-of-year assessment advice. The assessment advice mentors give is then formalized by a “portfolio assessment committee”. This committee holds the authority to either validate or overrule mentors’ advice, based on information from the portfolio and/or the second mentor. Generally, the committee adopts the mentors’ advice without further adjustments, and directly converts this into a final assessment.

### Participants

Within each program, we opted for mentors and mentees involved in the third year of the undergraduate programs, ensuring that they had experienced the full yearly cycle of low and high-stakes assessment at least twice. To contact participants, we used Qualtrics (Provo, Utah) survey software. Using this software, we emailed invitations and reminders to all eligible mentors to participate in this study. Based on convenience sampling, all who confirmed were invited for an interview hosted on Zoom (San Jose, California). Mentees were contacted via the university’s learning management system and group messages on social media (WhatsApp, Facebook) distributed by student representatives. All mentees who positively reacted to the invitation and were available for an interview were invited to participate. Mentees received a small digital gift card as a token of appreciation.

The final sample included 24 mentors of which 15 identified as women and nine as men. Mentors from both programs represented a range of professional backgrounds, including, but not limited to, basic scientist, physician, biomedical scientist, health scientist, psychologist, and educationalist. Eleven mentees participated with 10 identifying as women, one as man. Six mentees studied medicine and five studied biomedical sciences.

### Data collection

Our interview guide consisted of three sections: (1) open-ended questions about how participants regard their mentoring relationship, (2) exploratory questions guided by a vignette, and (3) questions about the combination of developmental support and assessment in mentoring. The open-ended questions in our interview guide were based on earlier work on mentoring and assessment [11,21,25,26,36]. The vignette contained a fictitious mentor-mentee conversation combining developmental support and assessment of the mentee. The first version of all interview materials was developed by LL and MT, consistently refined in dialogue with the larger research team, and piloted with a mentor and a mentee from the target population. Initial piloting resulted in small changes in the wording of some questions. A second pilot with another mentor did not result in further changes. The final versions of the interview guide and vignette can be found in Appendix 1 and Appendix 2.

All interviews were conducted and recorded by LL and MT between January 12 and March 30, 2022. Recurring discussions amongst the research team led us to conclude that after interviewing 24 mentors and 11 mentees we were able to build a rich understanding of how participants experience combining developmental support and assessment, and had reached data sufficiency [37,38].The interview recordings were transcribed verbatim and anonymized before further analysis.

## Ethical approval

Ethical approval was obtained from the Maastricht University Research Ethics Committee (UM-REC), file number: FHML-REC/2021/106, January 5, 2022.

## Data analysis

We used thematic analysis [31,39,40] to inductively identify, analyze, interpret, and display the data. LL reread the interview transcripts and process memos and drafted an inductive codebook. With this codebook, a set of five interviews was iteratively coded until no additional codes could be generated from the data. LL and PVG then discussed this codebook regarding completeness, omissions, and clarity, resulting in a refinement of the codebook and recoding of the initial five transcripts, supplemented by another five interviews. The entire research team checked and discussed the codebook. After the team agreed on this version of the codebook, LL coded all remaining transcripts. A list of all codes (translated from Dutch to English) can be found in Appendix 3.

The coded transcripts were used to draw up overviews per participant, based on a further clustering of our codes. With these clusters, we intended to briefly capture how participants experienced developmental support and assessment within mentoring, how they dealt with combining this in daily practice and to summarize participants’ most salient comments on support, assessment, and the relationship between those. All transcripts were re-read by LL, and the overviews were enriched with supporting quotes. We used the following eight clusters: (1) role of the mentor, (2) role of the mentee, (3) role of portfolio in mentoring, (4) mentoring goals, (5) meaning of feedback, (6) meaning of assessment, (7) opinion on design of programmatic assessment, and (8) opinion on having to combine support and assessment.

We used ATLAS.ti Version 22 (Scientific Software Development GmbH, Berlin, Germany) and Microsoft Excel 2016 (Microsoft Corporation, Redmond, Washington) to manage data throughout the analysis.

## Results

Undergraduate mentors and mentees experienced and dealt with combining developmental support and assessment in different ways. For some mentors, assessment was a well-integrated part of their mentoring. For others, it felt like an additional task, not belonging to what to them mentoring inherently entailed. Mentees expressed similar feelings; for some it was logical that their mentors assessed them, whereas others felt their mentor was not the right person to be entrusted with this task. So, for some participants combining developmental support and assessment worked well, whereas other mentor-mentee dyads experienced tension.

For this latter group we found tension to affect their relationship quality, dependence, trust, and the nature and focus of conversations. In some relationships, tension was intensified because of how programmatic assessment was implemented at the program level. Mentors who experienced tensions described different ways of alleviating these, which we categorized into three strategies: (1) transparency and expectation management; (2) distinguishing between developmental support and assessment; and (3) justifying assessment responsibility. Mentees mention similar approaches, but in less delineated strategies.

**Figure 1:**
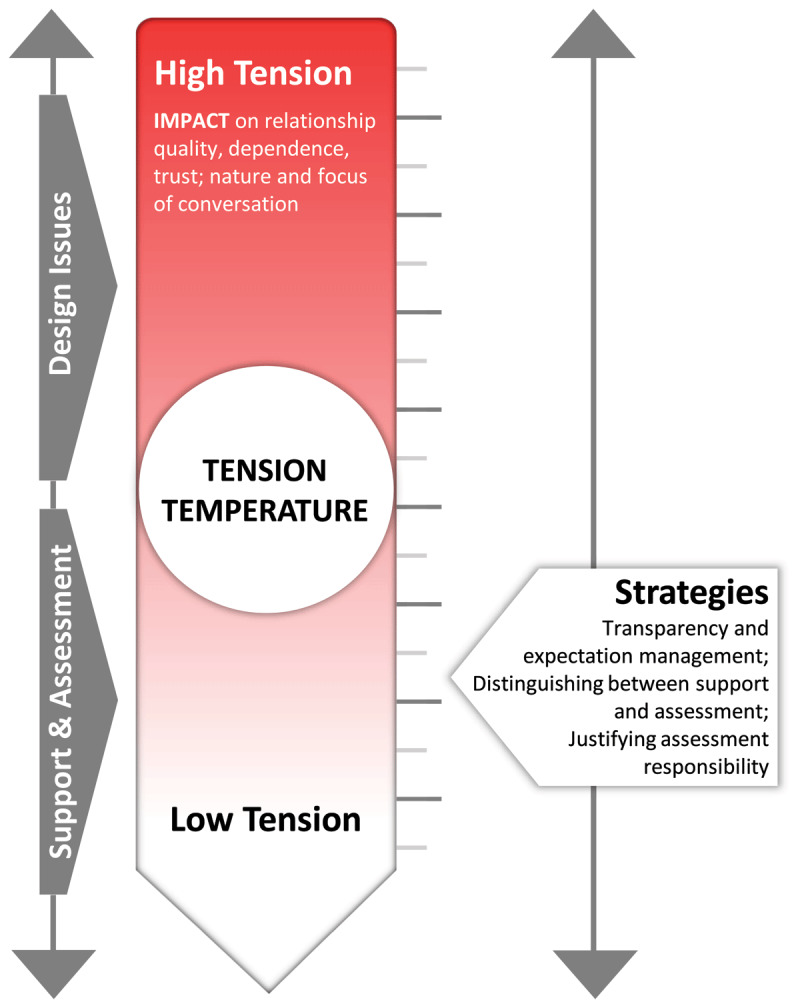
Tension Thermometer.

To visualize these results we introduce the metaphor of a “tension thermometer” (Figure 1). The factors displayed on the left and right side influence the experienced “tension temperature” in the central circle. Increased tension temperature can be alleviated with one or more of the strategies presented in the slider at the right of the figure. The remainder of this results section discusses the elements of the tension thermometer. To safeguard the anonymity of the mentees, the pronouns “they” and “them” were used for all mentees and their mentors to make descriptions less identifiable.

### Low tension

Mentors and mentees both stated that in well-functioning, informal relationships, where mentees were able to distinguish their mentors’ personal opinion from their professional assessment, there was no issue in being assessed by the mentor. This also was the case in relationships where mentees were doing well and assessment was positive. Some mentors stated that combining developmental support and assessment should not cause issues because they perceived this as an integrative part of their mentoring role: “It’s totally fine if you aim to develop a person and help them improve, and then also assess how that improvement is going.” [mentor8]. Some mentors considered themselves as the most appropriate or even the only person capable of properly assessing their mentees. Because of their longitudinal involvement, mentors got to know mentees on a personal level, witnessed their growth (or lack thereof), and could take into account mentees’ personal circumstances.

Some mentees agreed that due to the longitudinal nature of their relationship, their mentors saw their growth and development and knew their personal stories. This made the assessment feel more closely linked to their real life experiences. They felt that it was their mentor’s responsibility to make sure that assessment and developmental support could go hand-in-hand, and as long as mentees displayed an open attitude in their reflections, assessment had no negative impact on the mentoring relationship.

### Higher tension

In cases where mentors and mentees did experience a substantial degree of tension between developmental support and assessment, they mentioned different effects.

#### Impact on relationship, dependence, and trust

Mentors indicated a negative influence on the relationship with their mentees, such as reduced mentee openness or trust. They pointed out that in already strained relationships, feeling that they had to fail mentees imposed additional tension on that relationship, which could lead to a breach in trust. Mentors were also aware of the dependency mentees felt, and the double role they held in their eyes. “They’re still dependent of me, and even if I convince them that this will not make a difference, if I were them, I would not risk it either.” [mentor26] one mentor said when describing how open their mentees were to them.

Mentees confirmed that assessment could lead to a breach in trust and influenced the – at times fragile – dependency relationship between them and their mentors: “What if, as a medical student I start residency and I will be assessed by that same person again?” [mentee30]

#### Impact on nature and focus of conversations

Both mentors and mentees expressed that during mentor-mentee conversations, talking about competence or portfolio assessment (e.g., reaching a certain depth in the reflections or meeting a required number of portfolio items) often got in the way of talking about mentees’ personal stories or made conversations contrived. As one mentee said: “it is trying to objectify something very subjective.” [mentee35]. One of the mentors expressed: “It makes mentoring very artificial, everything is about assessment.” [mentor31]

### Higher tension because of design issues

Some mentors expressed that tensions they perceived were increased by issues inherent to the way programmatic assessment was implemented at the program level. An unintended consequence of the fact that the portfolio assessment committee frequently adopted mentors’ assessment advice one-to-one, was that mentors and mentees subsequently perceived this advice as the actual assessment, whereas ‒ technically ‒ mentors were only advising on the performance of mentees, not assessing them. Consequently, participants never spoke about “an assessment advice”, but about “assessing” or “being assessed”.

At the same time, mentors perceived their responsibilities to clash with those of other actors in the programmatic assessment system, for example, second mentors and portfolio assessment committees. A clash occurred when a portfolio assessment committee overruled mentors’ assessment, adjusted it on unclear grounds or without any explanation. At times, mentors also felt there were unspoken rules they were gauged against. As an exemplar, one mentor stated: “You are apparently not expected to grade more than half of your mentees as ‘above expectation’, because then they [the committee] will rein you in” [mentor37]. Mentors also felt scrutinized themselves; they felt held personally accountable when the portfolio of one of their mentees was not up to standards. This led some mentors to being stricter than necessary towards mentees. One mentor said, “I feel that I do that more to cover for myself, than for the development of the mentees.” [mentor31] when explaining why they required their mentees to extensively document everything in their portfolio.

### Strategies to alleviate tensions

Mentors shared several strategies they used to alleviate tensions. The way mentees handled tensions often manifested itself in less delineated strategies, but was noticeable in the way they dealt with their reflections and portfolio entries.

#### Transparency and expectation management

By communicating clear expectations and providing frequent and extensive feedback from the outset and throughout the year, mentors tried to make their assessment fair for their mentees. In doing so, they wanted to demonstrate that they were engaged with their mentees during the entire year. They already hinted on the outcome of their assessment during interim meetings: “I repeatedly tell mentees: ‘This is not up to expectations, and if that doesn’t improve I have to fail you [later this year]’.” [mentor20]. Mentors hoped this could prevent unsatisfactory portfolio grades altogether, or at the very least avoid surprises about a low grade later. If mentees were on track, mentors felt their feedback conversations throughout the year sufficed, and explicit conversations about the assessment were deemed unnecessary: “If everything is running smoothly and mentees are handling my feedback well, why should I still bother to talk about assessment explicitly?” [mentor20].

Mentees approved of this strategy of their mentors. They preferred mentors to be clear about what was expected, so they could ask for specific requirements. Also, they felt that as long as they were familiar with their mentors’ expectations in advance, it was fair if mentors would fail them after repeated feedback indicating that improvement was necessary but did not occur: “If they don’t tell you this, you still don’t know where exactly the areas of improvement are” [mentee27]. To deal with tension, mentees actually often appeared to become *less* transparent. They no longer genuinely reflected on their experiences, but wrote reflections on what they thought their mentors wanted to read when assessing their portfolio: “I make up and write down the emotions things can give me. I am not into feelings at all. I’m more of a thinker.” [mentee15]

#### Distinguishing between developmental support and assessment

Another strategy mentors applied was to distinguish between what they interpreted as supporting development (i.e., talking about the content of the reflections in the portfolios) and assessing development (i.e., checking the quality and quantity of those reflections in the portfolio). An example of this strategy is that mentors tried to minimize talking about assessment with their mentees as much as possible. They left it to the very end of a mentor meeting, after all personal matters were discussed, or completely removed assessment talk from meetings altogether, discussing it only via email instead: “I separate what is related to the portfolio assessment from my role as a mentor, because this is the only way to become a mentor. Only towards the end of a meeting I mention portfolios.” [mentor31].

Mentees agreed that tension would lessen when their mentors clearly distinguished between supporting and assessing development. In addition, for them, being assessed for the degree to which they showed personal or professional growth was acceptable. Their mentors should, however, not assess the ‘worthiness’ of the topics of their reflections and fail a student based on the content they were reflecting on. Related to that, a point stressed by multiple mentees was that mentors should take care not to sacrifice developmental support or a referral to a specialist for mentees that struggled (i.e., mentees with rather superficial reflections due to personal health circumstances should get a referral to a specialist, not an insufficient grade for their reflective skills).

In a way, mentees also tried to distinguish between support and assessment, albeit with a different effect in practice. Some of the mentees who experienced tension began to see their mentor as a kind of representative of the assessment program, disconnected from the intended development support goals. They saw mentor meetings as part of the assessment process instead: “At the end of the story, I don’t go to my mentor with my personal problems, I just go to them because it’s assessed and obligatory.” [mentee14]

#### Justifying assessment responsibility

Another strategy mentors used to alleviate at least a part of their experienced tension was justifying why they were the person engaged in both the developmental support and assessment of their mentees. Some explained to their mentees that assessment was obliged by the educational program. Others tried shifting the assessment responsibility onto someone else, like the second mentor or the portfolio assessment committee. One mentor explained this as follows: “I’m a coach, not an assessor, so I always use my second mentor. I say: ‘You have to do this for the second mentor.’ So do I hide behind that a little? Yes I do.”[mentor34]. Others tried siding with their mentees by complaining about the system together, or resorted to justifying their responsibilities in such a way that it seemingly minimized the effort required for, or importance attributed to, the assessment.

## Discussion

Our findings suggest that making undergraduate mentors responsible for both developmental support and assessment did not cause tension per se. In fact, for some mentors and mentees it fit well due to the longitudinal nature of a mentor-mentee relationship. In other mentor-mentee dyads, however, it did not provide a basis for a well-functioning relationship. Participants in the latter group indicated that it could generate tension, especially when the relationship between mentors and mentees was not optimal, when the assessment of mentees was unfavorable, or when the way programmatic assessment was designed hampered combining support and assessment. When assessment caused tension, the quality of the relationship and the degree of dependence and trust between mentors and mentees were impacted. Additionally, it changed the nature and content of the conversations between mentors and mentees. Mentors mentioned different strategies to alleviate tensions: they tried to be transparent towards their mentees about their expectations, they tried to distinguish between developmental support and assessment, or they tried to justify combining support and assessment. Mentees endorsed these strategies and showed related approaches to deal with tensions.

Comparing the experiences of our undergraduate mentors and mentees to those of participants in other research we noticed underlying similarities. The fact that combining developmental support and assessment is possible under certain conditions was also concluded elsewhere [11,26,41]. Valentine and Schuwirth [41], for example, concluded that assessment by a coach needs to be perceived as ‘fair’ (credible, transparent, fit for purpose, and accountable), for a learner to accept it and learn from it. We noticed participants in the current study reasoned along similar lines: some told us it made sense that there was an assessment component to the mentoring role, as mentors were the one to see mentees grow, or not. But this was only perceived as fair in well-functioning relationships (where the mentor was perceived as credible or accountable), or when it was clear to both parties what exactly was assessed ‒ and to what standards ‒, and why this was done by the mentor (in other words; when the assessment was perceived as fit for purpose and transparent). Atkinson and Watling [42] too noted that for feedback to be effective as developmental support, it not only needed to come from someone with whom mentees have developed a good relationship, by whom they felt respected, and who they perceived as credible and trustworthy, but that there also was a responsibility of the program to put into place effective learning and assessment structures, and to provide faculty development opportunities for mentors.

When interpreting the results from the point of the personal interpretative framework [27], we can indeed conclude that some mentors and mentees experience an incongruence between how they would like to mentor ‒ or be mentored – and what their professional context requires. This discrepancy between actual and preferred mentoring [43] could impact mentors’ professional self-understanding.

### Implications

Assigning undergraduate mentors to facilitate mentees’ development in a programmatic assessment context requires a commitment from all involved if we want to keep the tension-temperature low. The content of the program of assessment should be made clear: what is assessed, to what standards, and how [16,35,44,45,46,47]? For instance, how exactly will competence development and growth be assessed? Requiring a minimum number of reflections in a portfolio potentially increases the risk of artificial reflections and other strategic behavior of mentees towards mentors to pass the assessment. These mentees appear to engage in true reflection, but behave like “reflective zombies” [48,49,50] so that the portfolio no longer reflects their actual knowledge, skills, and attitudes, but rather operates as a form of impression management [16]. Developmental support and programmatic assessment should be implemented in such a way that they do not objectify mentees or prescribe standardized ways of reflection, but embrace diverse approaches to reflection [47,48], so that mentees are able to reflect authentically. Also, mentors should take care not to write off topics they deem unworthy of reflection [51].

To prepare mentors for possible tensions and support them in dealing with these tensions, assessment could be addressed during faculty development activities. Mentors could engage in peer consultation and discuss approaches of how to be clear on what they expect of their mentee and vice versa, how to provide feedback for mentees’ growth without making it feel like an assessment, and discussing the boundaries of their role and how to delineate these boundaries.

Additionally, on the program level, being able to combine developmental support and assessment requires a well-thought-out design, where the responsibilities of all involved do not interfere or contradict unintentionally [10,16]. Assessor training and frequent calibration sessions [12,19,52] would help mentors, portfolio assessment committees, and mentees [53] to co-construct a shared mental model on fair programmatic assessment.

### Strengths, limitations, and suggestions for further research

We deliberately made the decision to interview mentors and mentees from classroom-based programs. Because this undergraduate mentoring context in programmatic assessment has been explored to a much lesser degree in research than its graduate and clinical counterparts in HPE, it can add an additional perspective to the discussion. A drawback of this decision, however, is that due to its inherent contextual differences with workplace based learning it might be hard to fit this information into the puzzle of what is already known from previous research. We are convinced, however, that this classroom-based learning perspective adds value, as patterns in undergraduate mentor-mentee interactions and the expectations that become ingrained there form the basis of subsequent mentor-mentee relationships in the clinical workplace.

Due to our methodological choices we cannot be sure whether mentors and mentees in settings with a different programmatic assessment design also experience the tensions our participants brought forward. Considering this in light of the personal interpretative framework [27] and our earlier work on perceived discrepancies between actual and preferred mentoring approaches [43], however, we welcome the continuation of this research in different program contexts and with other groups of mentors or mentees. Further research into the experiences of mentors within programmatic assessment in undergraduate education contexts could, therefore, help strike a balance between developmental support and assessment in the mentor-mentee relationship. It is also worthwhile to explore the role of mentees in more depth: what agency do mentees have when being assessed by their mentors?

## Conclusion

Making undergraduate mentors responsible for both developmental support and assessment in a programmatic assessment context requires a well-implemented, clear program of assessment and unambiguous responsibilities laid out for all involved. When these conditions are not fully met, mentors and mentees will have to work harder for their relationship to function tension-free. This may involve tension alleviating strategies, but above all continuous discussion, calibration, fine-tuning, and agreement upon mutual expectations and commitments.

## Additional File

The additional file for this article can be found as follows:

10.5334/pme.1004.s1 Supplementary file 1. Appendix 1–3.

## Author Information

- Lianne M. Loosveld is an educational advisor and PhD candidate in faculty development at the School of Health Professions Education and the Department of Educational Development & Research, Faculty of Health, Medicine and Life Sciences, Maastricht, the Netherlands.
- Erik W. Driessen is a professor of medical education at the School of Health Professions Education and the Department of Educational Development & Research, Faculty of Health, Medicine and Life Sciences, Maastricht University, Maastricht, the Netherlands.
- Mattias Theys is a PhD candidate at the School of Health Professions Education and the Department of Educational Development & Research, Faculty of Health, Medicine and Life Sciences, Maastricht, the Netherlands.
- Pascal W. M. Van Gerven is an associate professor at the School of Health Professions Education and the Department of Educational Development & Research, Faculty of Health, Medicine and Life Sciences, Maastricht University, Maastricht, the Netherlands.
- Eline Vanassche is a tenure track professor at the Faculty of Psychology and educational Sciences at the KU Leuven Kulak, Belgium.
