# Supplementary material for: Combining Support and Assessment in Health Professions Education: Mentors’ and Mentees’ Experiences in a Programmatic Assessment Context
Source: Perspect Med Educ. 2023 Jul 7;12(1):271–81. doi: 10.5334/pme.1004 (PMC10327863; doi:10.5334/pme.1004)
Supplement: Supplementary file 1. — Appendix 1–3. [file pme-12-1-1004-s1.pdf]

Supplementary file 1:

Appendix 1 “interview guide”

INTERVIEW GUIDE

INTRODUCTION: Introduce yourself and let the participant introduce themselves.
Thank you again for taking the time to participate in this study. As stated in the information letter, this study addresses the current knowledge gap around how facilitating professional development and assessing interrelate during mentoring.

We will start with your own practice, then look at a vignette, and then come back to your own practice as well.
For Mentors: For your own practice, you can take the similarities or common denominator of your mentees in mind.

It is not my intention to judge you on your performance as a mentor/mentee, I am really looking for how you see this situation.

Is it ok for you if I record this interview? You can speak freely and critically if you wish; everything you say is confidential, and will only be used within the scope of my research on mentoring. If you mention names or specific situations I will anonymise these.

Do you have any questions at this moment?

I am now going to start the recording. [start recording]

1 Opening questions → daily practice

What does a regular mentor meeting actually look like for you?
How do you prepare for these conversations?
How does your mentor/mentee prepare for these conversations?
Who usually takes the lead in your mentor-mentee relationship? How does that make you feel?
What adjectives would you use to describe the connection between you and your mentee / your mentor?<br>So, for example open, distant, cozy, formal<br><br>Why exactly do you choose x? (and then purposefully explore the meaning of each chosen adjective)<br><br>// What kind of mentor-mentee relationship do you have?
When are you satisfied with relationship with your mentor/mentee?<br><br>(rephrase: what goals are you trying to achieve with your mentoring)
So, what is therefore needed for a successful relationship with your mentor/mentee? (ingredients, conditions, terms, etc.)

2 Questions after vignette

What did you think of the case?
If not mentioned: What do you think of the reaction of the mentor in this vignette?
If Benjamin was your mentee / If Ann was your mentor, how would you respond?
How do you expect the next meeting with the mentor/mentee to go?

3 Remaining questions → back to own daily practice

The vignette reflects several tasks of mentoring: guiding and assessing students.
What do you think about assessment as a mentor/mentee?
How important is it (for your mentor) to be part of this assessment process?
What role does that assessment play in your mentoring practice?
Let's follow up on that: how does that combination between supporting professional development and assessing the mentee work in your practice?<br>(Dichotomy, simultaneously, etc.)
Do you make that division explicit? Do you do this for yourself, and/or also to your mentee?
If yes: If you make this explicit (as e.g. in vignette), when and how do you make this clear: beforehand, when it comes up....
If not: How is assessment woven into the conversations you have with your mentor/mentees?
**OFF- the record questions:**<br>We've talked about this for a while now, did this reflection provide any take-aways for you on how you handle your mentorship? Are you going to take things back to practice?<br>What do you think now about our conversation?

Appendix 2 “interview vignette”

Setting:

This mentor-mentee meeting is taking place as the last meeting before the final mentor assessment. It is a face-to-face meeting at the office of the mentor, **Ann**.

The mentee (**Benjamin**) is doing okay regarding his investigator/scholar competency, passing most courses. His professional/organiser competency is up to par a little less, as he struggles with putting his deep reflections on paper and only has a very superficial portfolio.

Meeting:

As per usual, A(nn) and B(enjamin) started their Monday-afternoon conversation with an update on how their respective weekends were.

A: I went to the zoo this weekend with my children, my son just loves the giraffes, can’t get enough of them!

B: Oh, sounds fun! So, my weekend was not that great… My bike got stolen when I was out with friends, again…. If that wasn’t enough, my grandma also developed a fever yesterday and is getting tested for COVID today… I really hope she’s not infected but I am worried…

A: Oh, I’m sorry, that’s not good news, and I really hope you grandma can recover quickly.

B: Yeah thanks, I hope so too. But okay, I am here to talk about my practicals and upcoming exams so let’s do that.

After that, they talked for a bit about a practical that B failed and his upcoming exams. B now expresses his worries about his portfolio.

B: “well, I’m not sure about the number of experience cards I’ve made so far. Are you going to fail me on my professional/organiser competence when I don’t have enough cards?”

A: “Well, you can check the requirements for how many cards you need to make, but for me it’s not as much in the number of cards, but the depth of reflection in your cards that is still lacking. We’ve discussed this the last few meetings too, but until now I don’t see much progress to be honest.”

B: “Ok, well, but what should I do to correct that? As the final mentor assessment is rapidly coming closer, and I don’t know how to compensate for this?”

A: “Yes, the final mentor assessment is approaching indeed, and I do have to send in my final advice soon, so your concern is legitimate. Remember that at the end of last year we had this exact same conversation? I have seen you grow on many levels over the past three years and we always had constructive conversations, but we might need to revisit the heart of the matter. And such conversations are never the easiest to have. I mean, I’m wearing multiple hats in our mentor-mentee relationship; being the person who supports your personal and professional development, but also having to advise on your competencies and portfolio, and for the moment they are just not … [gets interrupted by B]”

B: “But you know me, right? We always had conversations on how I’m doing throughout these entire three years, and I always reflect on my experiences during our meetings.”

Appendix 3 “codes overview”

**Code group: Mentoring goals**
Development of: reflection, portfolio, student, study skills, Professional behaviour
Approachable, accessible, supportive
Ask how students are doing
Be of meaning to student /support/think along
Asking further / deeper questions
Explaining Maastricht University / academic world system
Befriending or not
Getting to know mentee as person
Honest and clear
Mentor learns from mentoring/mentee
Reassuring
Personal growth of mentee
Rapport building
Reflecting on past
Students participates in mentoring because of assessment (mentee goal)
Practical support in learning goals / card / reflections
Support Wellbeing
Talking about feelings
Trusted person

**Code group: Programmatic Assessment attitude**
Depersonification / scapegoating as rationale of portfolio
Assessment and supporting clash
Assessment directs student behaviour
P.A. creates Logistic/Admin/Bureaucracy/systems – type friction
Mentee doesn’t mind that mentor assesses too
mentee must document for external accountability
mentee should not be burdened with mentors’ portfolio opinion
Mentee writes portfolio towards/for/aimed at mentor
Mentor can assess
mentor content with system / portfolio
mentor does not use portfolio much
Mentor is needed to assess Portfolio
Mentor primarily assesses
Not assess but provide feedback
Mentor does not want to assess
Portfolio , assess and development are all the same / inextricably linked
Portfolio writing, reflection, professional behaviour are assessable competencies
Portfolio is responsibility of mentee
Separating between assessment and mentoring

Code group: Mentoring Relationship Type
Awkward
Distant
Driven by mentee
Driven by mentor
Formal/
Informal
Open/closed mentor
Open/closed student
Personal disclosure
Respectful
Technical/ instrumental

Code group: Role Perception
Advisor (or not)
Assessor “
Coach”
Mentor motivates student “
Objective vs subjective
Strict/ business-like or not”
Psychologist or not “
Mirror behaviour “
Supporting student with portfolio “

Codes without group
Mentor needs curriculum knowledge to be able to mentor
Mentor has multiple teaching roles
Programmatic assessment effect: Student is closed due to dependency relationship
